# Field and lab conditions alter microbial enzyme and biomass dynamics driving decomposition of the same leaf litter

**DOI:** 10.3389/fmicb.2013.00260

**Published:** 2013-09-03

**Authors:** Zachary L. Rinkes, Robert L. Sinsabaugh, Daryl L. Moorhead, A. Stuart Grandy, Michael N. Weintraub

**Affiliations:** ^1^Department of Environmental Sciences, University of Toledo, ToledoOH, USA; ^2^Department of Biology, University of New Mexico, AlbuquerqueNM, USA; ^3^Department of Natural Resources and the Environment, University of New Hampshire, DurhamNH, USA

**Keywords:** microbial biomass, extracellular enzyme, litter bag, decomposition, decomposer community, nutrients, turnover activity

## Abstract

Fluctuations in climate and edaphic factors influence field decomposition rates and preclude a complete understanding of how microbial communities respond to plant litter quality. In contrast, laboratory microcosms isolate the intrinsic effects of litter chemistry and microbial community from extrinsic effects of environmental variation. Used together, these paired approaches provide mechanistic insights to decomposition processes. In order to elucidate the microbial mechanisms underlying how environmental conditions alter the trajectory of decay, we characterized microbial biomass, respiration, enzyme activities, and nutrient dynamics during early (<10% mass loss), mid- (10–40% mass loss), and late (>40% mass loss) decay in parallel field and laboratory litter bag incubations for deciduous tree litters with varying recalcitrance (dogwood < maple < maple-oak mixture < oak). In the field, mass loss was minimal (<10%) over the first 50 days (January–February), even for labile litter types, despite above-freezing soil temperatures and adequate moisture during these winter months. In contrast, microcosms displayed high C mineralization rates in the first week. During mid-decay, the labile dogwood and maple litters in the field had higher mass loss per unit enzyme activity than the lab, possibly due to leaching of soluble compounds. Microbial biomass to litter mass (B:C) ratios peaked in the field during late decay, but B:C ratios declined between mid- and late decay in the lab. Thus, microbial biomass did not have a consistent relationship with litter quality between studies. Higher oxidative enzyme activities in oak litters in the field, and higher nitrogen (N) accumulation in the lab microcosms occurred in late decay. We speculate that elevated N suppressed fungal activity and/or biomass in microcosms. Our results suggest that differences in microbial biomass and enzyme dynamics alter the decay trajectory of the same leaf litter under field and lab conditions.

## INTRODUCTION

The mineralization of newly senescent leaf litter contributes approximately half of the annual carbon dioxide (CO_2_) efflux from soils in temperate deciduous forests (Schlesinger and Andrews, 2000). Complex interactions between litter quality and microbial communities regulate the magnitude of this carbon (C) flux and determine the trajectory of decay (Berg and McClaugherty, 2008). For instance, the same litter exposed to different microbial communities frequently displays pronounced differences in chemistry, even after substantial mass loss (Wallenstein et al., 2011; Wickings et al., 2011, 2012). Additionally, the complexity and diversity of litter chemical composition and its impact on microbial community function may explain why diverse plant litter mixtures often follow different decay trajectories than the average of the component species alone (Meier and Bowman, 2010). However, we lack detailed data on how microbial communities respond to labile and recalcitrant litter types at progressive stages of decomposition under field and laboratory conditions.

Decomposition rates of the same litter vary widely across terrestrial ecosystems. For instance, *Cornus *(dogwood) and *Quercus* (oak) mass loss can range from 50–75% to 25–55%, respectively, between field studies after one year of decomposition (Blair, 1988; Blair et al., 1992; Carreiro et al., 2000; Knoepp et al., 2005; Piatek et al., 2010). *Pinus* (pine) litter displayed highly variable decay rates over time after 5 years of field decomposition over 28 sites throughout North America (Gholz et al., 2000). Thus, it appears that site-specific factors influence microbial-substrate interactions and C flux patterns. We need to explain this underlying variability between field studies, especially how microbial behavior and decay rates of different litter types change in response to variations in climate and edaphic factors, to enhance the accuracy of decomposition models.

The influence of environmental factors on microbial dynamics and decomposition patterns is difficult to predict. For instance, soil temperature and moisture fluctuate seasonally in the field (Aerts, 1997; Liski et al., 2003), which influences microbial growth, as well as extracellular enzyme pools and activities (Baldrian et al., 2013). Variations in quality of plant litter, soil organic matter content and pH, and even wind velocity can alter microbial activity and decomposition rates (Berg and McClaugherty, 2008). Filamentous decomposers (i.e., actinomycetes and fungi) influence decomposition by physically integrating substrates that differ in C and nitrogen (N) availability, thus overcoming local nutrient limitation through translocation (Boberg et al., 2010). Therefore, the magnitude of decomposer responses to litter quality under field conditions is not consistent across sites due to the variable effects of biotic and abiotic factors on microbial-substrate interactions and C flux rates (Carreiro et al., 2000; Treseder, 2008; Snajdr et al., 2011).

In contrast to the influences of environmental conditions on decomposition in the field, laboratory microcosms isolate the intrinsic effects of litter and soil chemistry and microbial community from extrinsic effects of climatic variation. For instance, constant temperature and moisture conditions in the laboratory optimize CO_2_ production and decay rates (Risk et al., 2008). Laboratory microcosms also eliminate variable C and nutrient subsidies to microbial activity (Salamanca et al., 1998). For instance, microcosms prevent decomposers from using adjacent litter resources, exclude new microbial colonizers (i.e., fungi) from translocating nutrients from external sources during later stages of decay, and disregard interactions between plant roots and nutrients (Teuben and Verhoef, 1992). In addition, microcosms disrupt *in-situ* microbial consortia and networks, and homogenize microbial functional behavior that otherwise is spatially compartmentalized or heterogeneous under field conditions (Kampichler et al., 2001). Thus, laboratory microcosm studies describe interactions between microbial communities and leaf litter with much lower variability than in the field, and provide mechanistic information describing underlying processes of decomposition needed to refine decomposition models (McGuire and Treseder, 2009).

Traditional predictive models of litter decay assume that microbial communities are black boxes functioning in the same way in different environments (Bradford and Fierer, 2012). While useful in stable environments, these models do not sufficiently describe C flow or microbial dynamics under variable conditions (Schimel and Weintraub, 2003; Manzoni and Porporato, 2009). For instance, conventional models fail to capture how changes in microbial function impact C gains and losses throughout decay (Treseder et al., 2011). In contrast, mechanistic decomposition models that incorporate both decomposers and their enzymes as explicit drivers of decay predict C dynamics under variable conditions better than earlier models that simulated litter decay as a first-order process without microbial decomposers (Moorhead and Sinsabaugh, 2006; Moorhead et al., 2012). However, parameterizing mechanistic models is difficult due to the highly interactive and variable factors that influence microbial activity.

Our goal was to elucidate the microbial mechanisms underlying the variability associated with field litter bag studies by monitoring the decomposition dynamics of contrasting litter species in both field and lab settings. To accomplish this goal, we conducted parallel field and laboratory experiments using three litter species that varied in initial recalcitrance (dogwood < sugar maple < white oak) and an equal maple-oak mixture. We monitored mass loss, microbial biomass, and enzyme activity, and inorganic nutrient dynamics during decomposition. We established separate hypotheses for early (<10% mass loss), mid- (10–40% mass loss), and late (>40% mass loss) stages of decay:

*Early Decay: *We hypothesized that decomposition in early decay is regulated by the availability of water-soluble labile compounds. For instance, soluble substrates are preferentially consumed in fresh litter, primarily by decomposers with limited enzymatic capabilities. Thus, we expected that differences in mass loss between litter types in both the field and lab would increase with initial litter soluble content (dogwood > maple > mixture > oak). However, we predicted decay rates, microbial biomass to litter mass (B:C) ratios, and microbial demand for N and phosphorus (P) relative to C for all litter types to be greater in the lab than field, because of high water-soluble C availability in fresh litter combined with optimal temperature and moisture conditions for biological activity.

*Mid Decay: *We hypothesized that decomposition in mid-decay is regulated by the depletion of water-soluble C and labile nutrients, which would be reflected by increased extracellular enzyme activities targeting organic polymeric substrates. We expected microbial N and P demand to be greater in the lab than field, due to rapid depletion of nutrients from the limited amount of low-nutrient content soil used in the incubation. We predicted that nutrient limitation would decrease decay rates and B:C ratios for all litter types in the lab relative to the field, as decomposers cannot use external resources or translocate nutrients from adjacent litter in microcosms.

*Late Decay: *We hypothesized that the increasing relative proportion of lignin regulates decomposition in late decay. For instance, we used oxidative enzyme activity as a proxy for microbial breakdown of lignin. We expected that lab decomposition rates, B:C ratios, and microbial N and P demand would decrease in comparison to field incubations in response to the accumulation of nutrients in the lab microcosms. We predicted that lignolytic activity and biomass would be greater in the field, especially in the high lignin oak litter, due to increased fungal activity and the limited potential for excess nutrients to inhibit lignolytic activity.

Thus, we established separate hypotheses for early, mid-, and late decay to determine how environmental conditions and edaphic factors alter microbial-substrate interactions during decay.

## MATERIALS AND METHODS

### STUDY SITE

The study site was an oak-maple forest within the Oak Openings Region (N 41°33′, W 83°50′) of Northwest Ohio. Our study area was in the 1,500 ha Oak Openings Preserve Metropark. The mean annual temperature is 9.2°C and annual precipitation is 840 mm (DeForest et al., 2009). Soils have a low pH (~4.5) and are sandy, mixed, mesic Spodic Udipsamments (Noormets et al., 2008). Within the study area, we continually measured 30-min mean soil temperature [5 cm depth; CS107, Campbell Scientific Inc. (CSI), Logan, UT, USA] and soil water content [SWC (%); CS616, CSI] in the top 20 cm from probes buried within 500 m of a micrometeorological tower operated by the University of Toledo.

### LITTER COLLECTION

*Cornus florida *(dogwood),* Acer saccharum* (sugar maple) and *Quercus alba *(white oak) leaves were collected weekly in litter traps during October of 2009 within the study area. Litter was placed in paper bags, air dried, and maintained at room temperature at the University of Toledo. Using the same litter in both experiments minimized the potential for variation in litter chemistry to influence decomposition dynamics. Leaves (including petioles) were cut into 1 cm^2^ pieces to control how much litter mass went into each litter bag and to facilitate use in lab microcosms.

### FIELD LITTER BAG STUDY

Eight replicate 30 m^2^ plots were randomly selected (i.e., randomized block design) within the study area at least 150 m apart and within 500 m of the micrometeorological tower. Litter bags were constructed from nylon mesh measuring 15 × 15 cm (1 mm^2^ mesh size) and were filled with 5 g of either dogwood, sugar maple, white oak, or a 50% maple-oak mixture. Thirty litter bags (6 dogwood, 8 maple, 8 oak, and 8 of the mixture) were deployed in January 2010, into each of the 8 plots for a total of 240 litter bags. Litter bags were placed 3 m apart in direct contact with the soil surface and secured at their corners with 15 cm ground staples. Litter bags were collected during January 2010 (0 days), February 2010 (50 days), May 2010 (120 days), August 2010 (220 days), December 2010 (337 days), June 2011 (512 days), October 2011 (641 days), and May 2012 (849 days). Dogwood litter bags were harvested only six times (June 2011 and May 2012 were excluded) due to its rapid decomposition. Destructive harvests included analyses for mass loss, extracellular enzyme activities, microbial biomass-C (MB-C), dissolved organic C (DOC), ammonium (${NH}_{4}^{+}$), nitrate (${NO}_{3}^{-}$), and phosphate (${PO}_{4}^{3-}$), described below.

### LABORATORY INCUBATION

The soil used in the laboratory incubation was a sandy soil low in C (0.6 ± 0.01%) and nutrient content, collected from a 10 m^2^ area where the field litter bag study was conducted. Soil cores were taken from the top 5 cm (the depth with the highest biological activity), sieved (2 mm mesh) to remove coarse debris and organic matter, thoroughly mixed, and pre-incubated for 1 month in a dark 20°C incubator at 45% water-holding capacity (WHC). This is the water content that maximizes microbial respiration (Rinkes et al., 2013). The pre-incubation allowed microorganisms to acclimate to the conditions of the experiment and to metabolize labile soil C.

A 376-day laboratory incubation was established in 473-ml canning jars using the same litter as the field study (see above). Nylon mesh 6 cm × 6 cm litter bags (1 mm^2^ mesh size) were constructed to lay flat inside each canning jar. Each litter bag contained 1 g litter and 1 g dry soil, which was used as a microbial inoculum to enhance colonization. Treatment jars included a litter bag placed in the middle of 99 g dry soil adjusted to 45% WHC. Soil-only control jars contained 100 g dry soil adjusted to 45% WHC. Eight sets of 32 litter bag + soil jars (each set with four jars each of dogwood, maple, oak, and the mixture) and four sets of soil-only control jars (each set with four replicate jars), were incubated together. Jars were kept in a dark 20°C incubator with lids left loosely covered, which minimized water loss but allowed gas exchange. Jars were weighed initially and deionized water was added gravimetrically on a weekly basis to replace water lost to evaporation.

Litter bags were destructively harvested after 0, 2, 34, 99, 161, 230, 312, and 376 days of decomposition. Adhering soil particles were removed from the litter with a 2-mm brush before analyses for mass loss, enzyme activities, MB-C, DOC, ${NH}_{4}^{+}$, ${NO}_{3}^{-}$, and ${PO}_{4}^{3-}$. Soil-only controls were destructively harvested less frequently than litter treatments, but respiration rates were monitored frequently in both litter bag + soil treatments and soil-only controls.

### MICROBIAL RESPIRATION AND MASS LOSS

Respiration was quantified in the lab by measuring jar headspace CO_2 _ concentrations with a Li-820 infra-red gas analyzer (LI-COR Biosciences, Lincoln, NE, USA) according to the manufacturer’s protocol. Jars were vented, sealed in canning jars with septae (No.:224100-181 Wheaton grey butyl stoppers) installed in the lids, and incubated at 20°C for minutes (early decay) to hours (late decay). Respiration was measured at 0, 1, 2, 3, 5, 7, 8, 25, 43, 53, 78, 99, 139, 161, 230, 259, and 376 days of incubation. Cumulative C mineralization was calculated for the laboratory experiment by determining mean rates between measurements and interpolating over time. The initial C content ranged from 40 to 45% for all litter types and was used to calculate overall C losses. A 2400 Series II CHNS/O Analyzer (PerkinElmer, Waltham, MA, USA) was used to obtain the litter C content. In addition, litter mass loss was calculated as the difference in dry weight before and after incubation in both the field and lab.

### MICROBIAL BIOMASS AND NUTRIENTS

To extract litter samples for DOC, dissolved inorganic N (DIN), and dissolved inorganic P (DIP), 15-ml aliquots of an aqueous 0.5 M solution of potassium sulfate were added to each homogenized sample and agitated at 120 rpm on an orbital shaker for 1 h. Samples were vacuum filtered through Pall A/E glass fiber filters and frozen until analysis. Replicate samples were also fumigated with chloroform to quantify MB-C using a modification of the chloroform fumigation-extraction method (Brookes et al., 1985) described by Scott-Denton et al. (2006). Ethanol-free chloroform (2 ml) was added to 0.25 g (wet weight) of litter (including 3-litter free blank flasks) and incubated at room temperature for 24 h in a stoppered 250-ml Erlenmeyer flask. Following incubation, flasks were vented in a fume hood for 30 min and extracted as described above. Fumigated extracts were analyzed for total DOC on a Shimadzu total organic carbon (TOC-VCPN) analyzer (Shimadzu Scientific Instruments Inc., Columbia, MD, USA) using the non-purgeable organic C manufacturer’s protocol, which eliminates inorganic C prior to analysis. MB-C was calculated as the difference between DOC extracted from fumigated and non-fumigated samples. No extraction efficiency constant (*k*_EC_) was applied because it is unknown for these samples.

Colorimetric microplate assays of the unfumigated sample extracts were used to analyze ${NH}_{4}^{+}$, ${NO}_{3}^{-}$ and ${PO}_{4}^{3-}$ concentrations. ${NH}_{4}^{+}$ concentrations were measured using a modified Berthelot reaction (Rhine et al., 1998).${NO}_{3}^{-}$ was determined using a modification of the Griess reaction (Doane and Horwath, 2003), which involves the reduction of nitrate to nitrite for colorimetric determination. ${PO}_{4}^{3-}$ was analyzed following the malachite green microplate analysis described by D’Angelo et al. (2001). Absorbance values were determined on a Bio-Tek Synergy HT microplate reader (Bio-Tek Inc., Winooski, VT, USA) according to the manufacturer’s protocol.

### ENZYME ASSAYS

Fluorimetric enzyme assays were conducted using procedures defined by Saiya-Cork et al. (2002). We measured β-1,4-glucosidase (BG), β-1,4-*N*-acetyl-glucosaminidase (NAG), leucine amino peptidase (LAP), and acid phosphatase (Phos) activities in 96-well microplates using methyl umbelliferyl linked fluorimetric substrates. BG hydrolyzes glucose from cellulose oligomers, especially cellobiose; NAG (a.k.a. chitinase) hydrolyzes *N*-acetyl glucosamine from chitin and peptidoglycan-derived oligomers; LAP hydrolyzes leucine and other amino acids from peptides; and Phos hydrolyzes phosphate from phosphate monoesters such as sugar phosphates. These enzymes were selected because they catalyze terminal reactions that release assimilable nutrients from organic C, N, and P sources (Sinsabaugh and Follstad Shah, 2012).

Slurries were made using 0.25 g of wet litter homogenized with 50 mM sodium acetate buffer (pH 4.5) using a Biospec Tissue Tearer (BioSpec Products, Bartlesville, OK, USA) according to the manufacturer’s protocol. For the lab litter bags, adhering soil particles were removed from the litter using a 2-mm brush before addition to the slurry. We used a 200 μM substrate solution (4-MUB-β-D-glucoside for BG; 4-MUB-*N*-acetyl-β-D-glucosaminide for NAG; L-leucine-7-amino-4-methylcoumarin for LAP; and 4-MUB-phosphate for Phos) to ensure saturating substrate concentrations (German et al., 2011). After substrate addition, microplates were incubated at 20°C in darkness for at least 2 h.

High-throughput colorimetric assays were conducted in 96-well microplates for phenol oxidase (Phenox), which is a lignin-degrading enzyme, using 2,2′-azino-bis-3-ethylbenzothiazoline-6-sulfonic acid (ABTS) as a substrate (Floch et al., 2007). Assay wells included 200 μl aliquots of soil slurry followed by the addition of 100 μl of 1 mM ABTS. Negative controls received 200 μl of buffer and 100 μl ABTS and blank wells received 200 μl of soil slurry and 100 μl of buffer. Plates were incubated at 20°C in darkness for at least 2 h.

### DATA ANALYSIS

The relationship between the remaining leaf litter mass and time (days) was fit to a simple negative exponential model of decay (Olson, 1963). Decay rate constants were determined from the equation *X*_t_ = *X*_0_e^(^^-^**^kt^^)^ where *X*_t_ is the mass remaining at time = *t*, *X*_0_ is the initial mass, *t* is time in days and *k* is a decay rate constant in days^-^^1^. Decay rate coefficients (*k* values) were calculated at different stages of decay for both the field and lab studies and *k* values for each stage were compared with a one-way analysis of variance (ANOVA) including litter type as the fixed effect.

Ratios of microbial biomass to litter mass (B:C) were compared at different stages of decay in both the field and lab studies using a two-way ANOVA with stage and litter type as fixed effects. B:C ratios were examined in early decay (January and February 2010 field harvests and days 0–2 in lab), mid-decay (May 2010 through December 2010 field harvests, and days 34–161 in lab), and late decay (June 2011 through May 2012 field harvests, and days 230 through 367 in the lab).

Enzyme activities were expressed as μmol reaction product hour^-^^1^ g dry litter^-^^1^. These values were subsequently integrated over all sample dates according to Sinsabaugh et al. (2002) to derive cumulative enzyme activities and calculate turnover activities. Turnover activity, or the cumulative amount of enzyme activity per unit mass loss, provides a basis for comparing microbial allocation toward extracellular enzymes and the efficiency of decomposition (higher turnover activity = lower mass loss per unit enzyme activity) across different litter types. In brief, cumulative enzyme activity was calculated by multiplying the average activity of an enzyme between harvest dates over a specific time period. Turnover activity for BG, NAG, LAP, Phos, and Phenox was calculated by dividing cumulative enzyme activities by total mass loss for each sample replicate over time. Mean turnover activity (mol) was calculated from the turnover activities for all replicates (*n* = 8 in field, *n* = 4 in lab). Separate two-way ANOVA’s were performed at each stage of decay with litter type and enzyme as fixed factors. Turnover activities were examined in early decay (January through February 2010 field harvests and days 0–2 in lab), mid-decay (May 2010 through December 2010 field harvests, and days 34–161 in lab), and late decay (June 2011 through May 2012 field harvests, and days 230 through 367 in the lab) and overall.

We used BG:(NAG + LAP) and BG:Phos ratios as indicators of microbial demand for C relative to nutrients, and (NAG + LAP):Phos ratios as an indicator of microbial demand for N relative to P in both studies at each decay stage. Ratios of these C:N:P acquiring enzyme activities broadly converge on a 1:1:1 ratio across a wide range of sample types and ecosystems, and deviations from this ratio indicate relatively more or less decomposer demand for C, N, or P (Sinsabaugh et al., 2008). C:N:P ratios were examined in early decay (January and February 2010 field harvests and days 0–2 in lab), mid-decay (May 2010 through December 2010 field harvests, and days 34–161 in lab), and late decay (June 2011 through May 2012 field harvests, and days 230 through 367 in the lab) and overall.

In addition, data from the field litter bag study were analyzed using a mixed-model two-way multivariate ANOVA (MANOVA) with day and litter type as fixed effects and subplot (block) as a random effect. Mass loss, ${NH}_{4}^{+}$, ${NO}_{3}^{-}$, ${PO}_{4}^{3-}$, BG, NAG, Phos, and Phenox means were compared for the six harvest dates for which we have data for all litter types (including dogwood). The block effect was not significant overall (Wilk’s λ = 0.89,* P* = 0.73) or for each individual response variable, therefore the MANOVA was rerun without block as a factor (Scheiner and Gurevitch, 1993) and results from this final analysis were reported. For the laboratory incubation, mass loss, ${NH}_{4}^{+}$, ${NO}_{3}^{-}$, ${PO}_{4}^{3-}$, BG, NAG, and Phos means were compared using a two-way MANOVA with day and litter type as fixed effects.

Differences among groups were considered significant if *P* í 0.05. Differences between groups were compared using Tukey multiple comparison post-hoc tests. Enzyme activities were log-transformed to meet assumptions for normality and homogeneity of variance (Levene’s test). Data were analyzed using SPSS Statistics version 17.0.

## RESULTS

### FIELD STUDY – CLIMATE AND MASS LOSS RELATIONSHIPS

**FIGURE 1:**
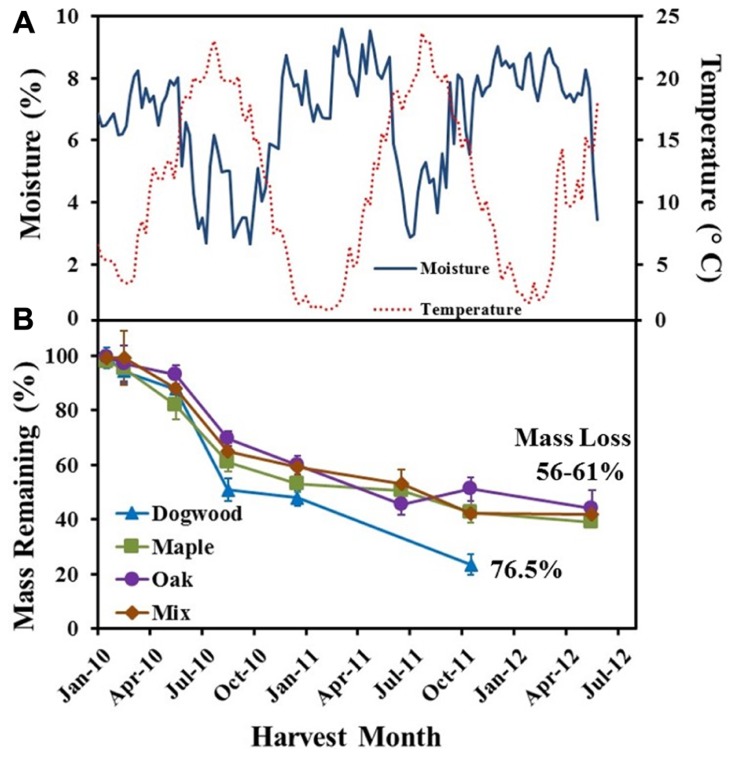
**(A)** Soil temperature (°C) and soil water content (%) weekly averages and **(B)** % mass remaining for dogwood, maple, oak, and the maple-oak mixture over a 2 1/2 year field litter bag study. Error bars show the standard error of the mean (*n* = 8).

Average weekly soil temperatures ranged from 1 to 24°C, while mean weekly SWC ranged from 2 to 10% over the course of the field study (**Figure 1A**). Over the initial 2 months of decomposition, litter mass loss was low for dogwood (5.8 ± 3.7%), maple (4.7 ± 3.2%), oak (5.4 ± 3.6%), and the maple-oak mixture (1.7 ± 1.3%; **Figure 1B**). During this time, soil temperatures averaged 5.6 ± 0.13°C and SWC averaged 6.7 ± 0.16%. Dogwood, maple, oak, and the litter mixture lost 37 ± 6.6%, 22.9 ± 6.5%, 23.4 ± 4.5%, and 24.6 ± 6.3% of their original masses, respectively, during May through August 2010 (**Figure 1B**). Mass loss then decreased for dogwood (27.3 ± 6.1%), maple (19.1 ± 5.5%), oak (20.4 ± 4.6%), and the mixture (22.9 ± 3.5%) between August 2010 and October 2011 (**Figure 1B**). SWC was highly variable but generally increased during the winter and decreased during the summer (**Figure 1A**). Mass loss was <7% for maple, oak, and the mixture between October 2011 and May 2012.

Decay rate coefficients were higher for dogwood than the other litter types during mid- and late decay and overall in the field (**Table 1**). In addition, decay rate coefficients were higher for dogwood and maple in the field than lab during mid-decay (**Table 1**). Overall, mass loss was greatest for dogwood (76.5 ± 2.7%) followed by maple (60.9 ± 1.9%), the maple-oak mixture (58 ± 2.6%), and oak (56.1 ± 7.5%). This resulted in a significant litter type effect on mass loss (*P* < 0.01; *F *= 5.69).

**Table 1:** Decay rate coefficients (*k*) for dogwood, maple, oak, and the maple-oak mixture during early, mid-, and late decay, and over the entire duration of the 849-day (641-day for dogwood) field and 376-day laboratory litter bag studies.

	Early		Mid		Late		Overall			
	Field 0–50 days	Lab 0–34 days	Field 50–337 days	Lab 34–161 days	Field 337 days - end	Lab 161–376 days	Field		Lab	
	*k* (day^ - 1^)		*k* (day^ - 1^)		*k* (day^ - 1^)		*k* (day^ - 1^)	*R*^ 2^	*k* (day^ - 1^)	*R*^ 2^
Dogwood	0.0012 a	0.0068 b	0.0026 d	0.0017 d	0.0020 e	0.0007 g	0.0023 h	0.97	0.0016 i	0.85
**Maple**	0.0010 a	0.0048 c	0.0020 e	0.0019 d	0.0006 f	0.0007 g	0.0011 g	0.91	0.0015 i	0.89
**Oak**	0.0011 a	0.0049 c	0.0016 e	0.0023 d	0.0006 f	0.0010 g	0.0009 g	0.88	0.0018 i	0.92
**Mix**	0.0005 a	0.0047 c	0.0018 e	0.0022 d	0.0007 f	0.0007 g	0.0010 g	0.93	0.0016 i	0.89

*Decay coefficients for each decay stage in the field and lab were compared among the different litter types with 1-way ANOVAs and differences between litter types were compared with Tukey's post-hoc tests, when necessary. Lowercase letters designate significant differences between litter types within each decay stage and study. *R*^2^ values represent the variance explained by the regression model and all *P* values are <0.05.*

### LAB INCUBATION-MASS LOSS

In the lab, decay rate coefficients were significantly higher for dogwood than the other litter types during early decay, but similar among all litter types over the rest of the study and overall (**Table 1**). In addition, C mineralization rates for all litter types peaked within the first week and were higher on day 2 in dogwood than the other litter types (*P* < 0.01 for all; data not shown). Dogwood, maple, oak, and the litter mixture lost 20.7 ± 0.6%, 15.9 ± 0.3%, 16.8 ± 0.5%, and 14.8 ± 0.5% of their original masses, respectively, between days 0 and 34. Over the next 127 days, mass loss was 15.6 ± 0.9% for dogwood, 18.3 ± 1.5% for maple, 21.3 ± 1.5% for oak, and 20.7 ± 1.2% for the mixture. Over the remainder of the incubation, dogwood, maple, and the mixture lost approximately 9% of the original mass, while oak lost 12.9 ± 3.2%.

### BIOMASS DYNAMICS

**FIGURE 2:**
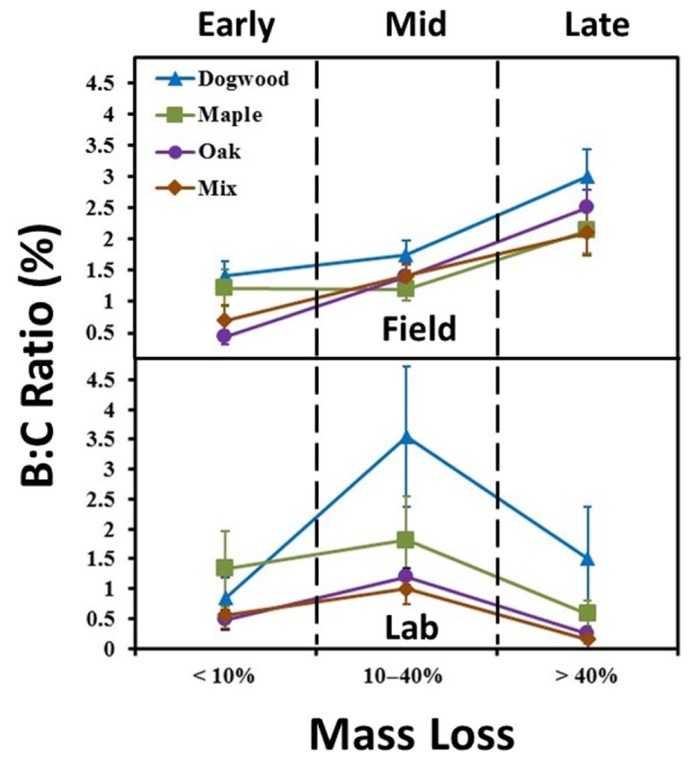
**Biomass to litter mass (B:C) ratios for the field and laboratory incubations.** Ratios were examined in early decay (January and February 2010 field harvests and days 0–2 in lab), mid-decay (May 2010 through December 2010 field harvests, and days 34–161 in lab), and late decay (June 2011 through May 2012 field harvests, and days 230 through 367 in the lab). Error bars show the standard error of the mean (*n* = 8 for field and *n* = 4 for lab per harvest). B:C ratios changed over time (*P* < 0.05) and were higher in late decay in the field and during mid-decay in the lab for all litter types.

In the field, B:C ratios for all litter types in late decay were significantly higher than values in early or mid-decay (*P* < 0.02 for all; **Figure 2**). Maximum B:C ratios in the field ranged from 2 to 3% (**Figure 2**). B:C ratios in the lab were significantly higher in mid-decay than late-decay for all litter types (*P* < 0.05 for all; **Figure 2**). Maximum B:C values ranged from 1 to 3.5% in the lab (**Figure 2**).

### INORGANIC NUTRIENTS

**FIGURE 3:**
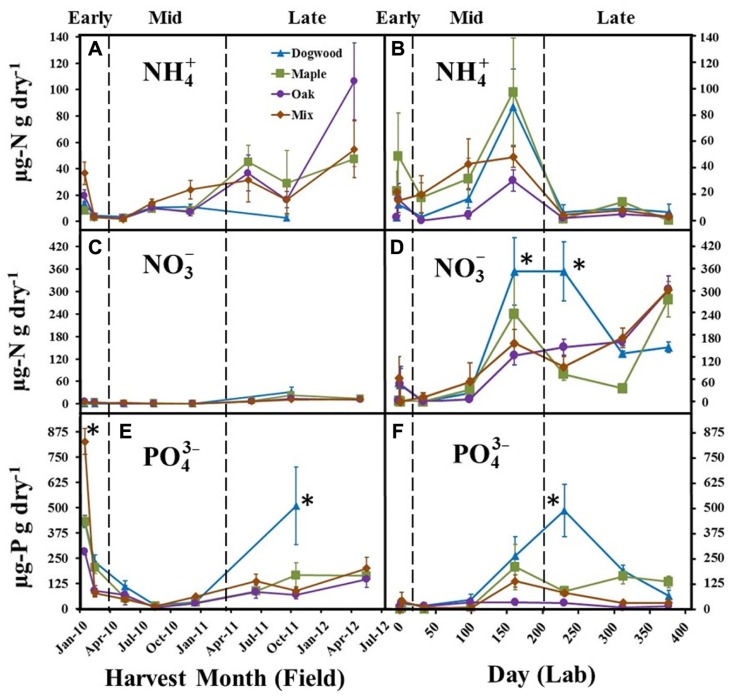
**${NH}_{4}^{+}$, ${NO}_{3}^{-}$, and ${PO}_{4}^{3-}$ concentrations for dogwood, maple, oak, and the maple-oak mixture in the field litter bag study (A, C, E) and laboratory incubation (B, D, F) during early (>10% mass loss), mid-(10–40% mass loss), and late (>40% mass loss) decay.** Error bars show the standard error of the mean (*n* = 8 for field and *n* = 4 for lab). Asterisks (*) denote significant differences (*P* < 0.05) between litter types on a harvest date.

${NH}_{4}^{+}$ significantly changed over time in both the field (*P* < 0.01; *F* = 4.57) and lab (*P* < 0.01; *F* = 10.29). ${NH}_{4}^{+}$ decreased during early decay for all litter types and remained low (<20 μg N g dry litter^-^^1^) through mid-decay in the field (**Figure 3A**), but increased during late decay for maple, oak, and the maple-oak mixture. In the lab, ${NH}_{4}^{+}$ decreased during early decay and then increased during mid-decay for all litter types, but then decreased during late-decay and remained low (<20.0 μg N g dry litter^-^^1^) between days 230 and 376 (**Figure 3B**).

Extractable ${NO}_{3}^{-}$ was low (<25.0 μg N g dry litter^-^^1^) for all litter types throughout the field study (**Figure 3C**). In contrast, ${NO}_{3}^{-}$ increased quickly during mid- and late decay in the lab for all litter types (**Figure 3D**) and was significantly higher in dogwood compared to most other litters on days 161 and 230, which resulted in a significant litter type by day interaction for ${NO}_{3}^{-}$ (*P* < 0.01; *F* = 3.22).

${PO}_{4}^{3-}$ significantly decreased for all litter types during early decay in the field (**Figure 3E**). ${PO}_{4}^{3-}$ remained low for all litter types throughout mid-decay, but increased during late decay. ${PO}_{4}^{3-}$ was significantly higher in the mixture during the initial harvest and in dogwood during the October 2011 harvest than other litter types (*P* < 0.01 for all; **Figure 3E**). In the lab, ${PO}_{4}^{3-}$ concentrations increased for most litter types during mid- and late decay and were significantly higher in dogwood on day 230 than other litter types (*P* < 0.01 for all; **Figure 3F**). Overall, there were significant litter type by day interactions on ${PO}_{4}^{3-}$ in the field (*P* < 0.01; *F* = 7.49) and lab (*P* < 0.01; *F* = 4.37).

### ENZYME ACTIVITY

**FIGURE 4:**
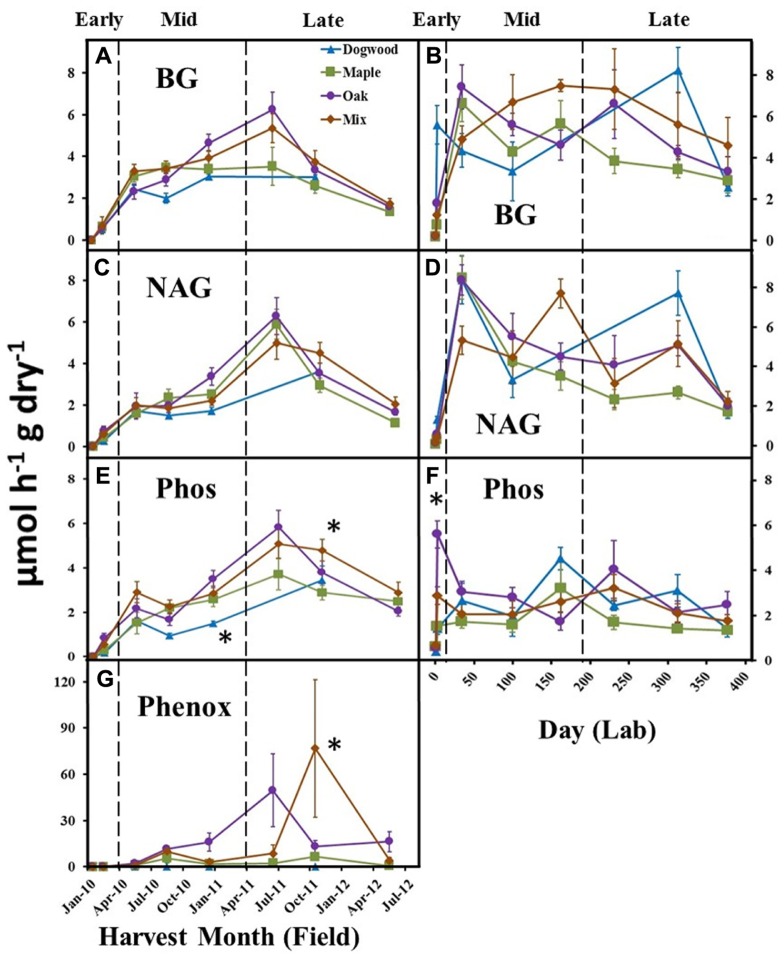
**BG, NAG, Phos, and Phenox activities over time for dogwood, maple, oak, and mixed litter in the field litterbag study (A, C, E, G) and laboratory incubation **(B**, **D**, **F)** during early (<10% mass loss), mid- (10–40% mass loss), and late (>40% mass loss) decay**. Phenox activity was not detected in the lab. Error bars show the standard error of the mean (*n* = 8 for field and *n* = 4 for lab). An * designates differences between litter species at each harvest date.

In the field, BG activities peaked during late decay for maple, oak, and their mixture (**Figure 4A**). BG activity increased following the initial harvest in the lab (**Figure 4B**) and was significantly higher in dogwood compared to other litter types on day 230 (not shown), which resulted in a significant litter type by day interaction (*P* < 0.01; *F* = 5.45). NAG activity increased following the initial harvest in the field and lab, but did not differ between litter types in the field (**Figures 4C, D**). NAG activity was higher in dogwood than other litter types on day 230 (not shown) in the lab, which resulted in a significant litter type by day interaction (*P* < 0.01; *F* = 2.46). Phos activities peaked for maple, oak, and the mixture during late decay and were significantly higher in oak than dogwood during the December 2010 harvest and the mixture compared to maple during the October 2011 field harvest (**Figure 4E**). Phos also increased rapidly following the initial harvest in the lab and was significantly higher in oak than other litter types on day 2 (**Figure 4F**). Overall, there were significant litter type by day interactions on Phos in the field (*P* = 0.01; *F* = 2.73) and lab (*P* < 0.01; *F* = 2.30). LAP activity remained relatively low (<0.3 μmol h^-^^1^ g dry litter^-^^1^) for all litters throughout both studies (data not shown).

Phenox activities were low in dogwood and maple throughout the field study and were significantly higher in mixed litter compared to other litter types during the October 2011 harvest (*P* < 0.01 for all; **Figure 4G**). Phenox activity was also elevated in oak during late decay (June 2011 and May 2012) (**Figure 4G**). Due to increasing Phenox activity in oak and the maple-oak mixture, there was a significant litter type by day interaction (*P* < 0.01; *F* = 4.90) on Phenox activity in the field. Phenox was not detectable at any time during the lab incubation.

### TURNOVER ACTIVITY

**FIGURE 5:**
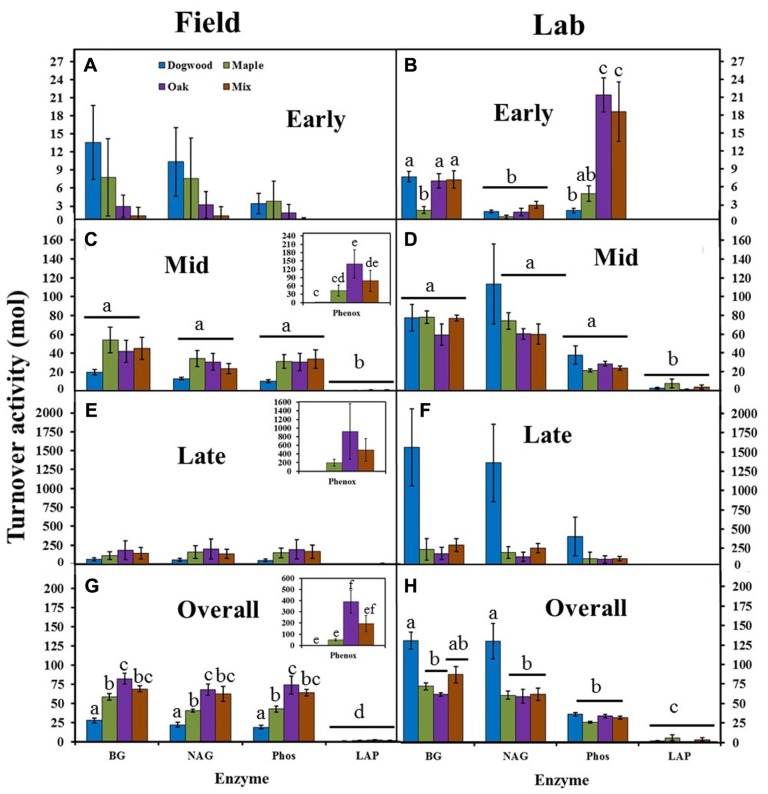
**BG, NAG, LAP, Phos, and Phenox turnover activities (mol) for dogwood, maple, oak, and the maple-oak mixture in the field litter bag study and laboratory incubation during early (A, B), mid- (C, D), and late (E, F) decay, and overall (G, H).** No Phenox activity was detected in the lab or during early decay in the field. Turnover activities (high turnover activity = high enzyme activity per unit mass loss) were compared with separate two-way ANOVA’s (field and lab) during each decay stage and lowercase letters designate significant differences within and across enzymes for each study. Error bars show the standard error of the mean (*n* = 8 for field and *n* = 4 for lab). No significant differences between enzymes or litter types occurred during early decay in the field. Phenox turnover activity was significantly higher than most other enzymes during late decay in the field, while dogwood turnover activity was significantly higher than other litter types during late decay in the lab.

Turnover activities were low (<15 mol) and did not differ between litter types or enzymes in the field during early decay (**Figure 5A**). In the lab, turnover activities were also low (<25 mol), but were significantly higher in oak and the mixture than other litters for Phos (*P* < 0.01; **Figure 5B**). BG and NAG turnover activities were >60 mol in the lab, but <50 mol in the field for all litter types during mid-decay (**Figures 5CD**). Turnover activity differed between enzymes (*P* = 0.01; *F* = 3.44) and litter types (*P* = 0.04; *F* = 2.87) during late decay in the field due to higher Phenox and lower dogwood turnover activities compared to most other enzymes and litter types (**Figure 5E**). However, turnover activity for dogwood >maple, oak, and their mixture in the lab (**Figure 5F**), which produced a significant litter type effect (*P* = 0.03; *F* = 3.20). Turnover activities were as much as an order of magnitude higher in the lab than field during late decay (**Figures 5E, F**). Overall, turnover activity in dogwood < maple < oak for BG, NAG, and Phos in the field (*P* < 0.04 for all; **Figure 5G**). Maple decomposition required 2.2× more BG per unit mass loss, 1.8× more NAG, and 2.1× more Phos than dogwood, while oak and the maple-oak mixture required approximately 1.5× more BG, NAG, and Phos than maple. In the lab, turnover activity in dogwood > maple and oak for BG and NAG (*P* < 0.02 across all enzymes), but did not differ between litter types for Phos (**Figure 5H**). Dogwood decomposition required approximately 2× more BG and NAG than other litter types.

Phenox turnover activity was undetectable in early decay and significantly higher than most other enzymes during mid-and late decay and overall in the field (*P* < 0.01 across all enzymes; **Figures 5A, C, E, G**). Overall, Phenox was 2–4× higher in oak compared to dogwood, maple, and the maple-oak mixture. LAP turnover activity was significantly lower than most other enzymes and did not differ between litter types in both the field and lab (**Figure 5**). Differences in enzyme turnover activities between litter types resulted in significant enzyme type by litter type interactions during mid-decay (*P* < 0.01; *F* = 2.39) and overall in the field (*P* < 0.01; *F* = 6.63) and during early decay (*P* < 0.01; *F* = 8.75) and overall in the lab (*P* < 0.01; *F* = 3.56).

### ENZYME ACTIVITY RATIOS

**FIGURE 6:**
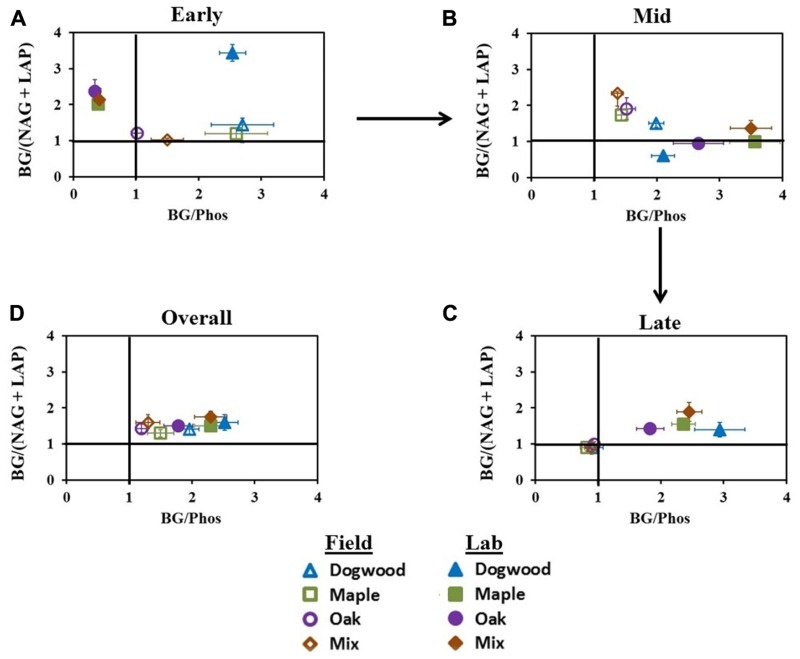
**C:N and C:P acquisition ratios at different stages of decay during the field and lab incubations.** C:N:P acquisition converges on a 1:1:1 ratio, therefore BG:(NAG + LAP) or BG:Phos ratios deviating from 1 in each scatterplot indicate differences in relative microbial demand for C and nutrients. Ratios were examined in **(A)** early decay (January through February 2010 field harvests and days 0–2 in lab), **(B)** mid- decay (May 2010 through December 2010 field harvests, and days 34–161 in lab), and **(C)** late decay (June 2011 through May 2012 field harvests, and days 230 through 367 in the lab) and **(D)** overall.

In early decay, C:N acquisition ratios were >1 for all litter types in both studies. C:P acquisition ratios were also >1 for all litter types in the field, but in the lab, C:P acquisition ratios were <0.5 for maple, oak, and their mixture and >1 for dogwood (**Figure 6A**). Additionally, N:P ratios were <1 (data not shown) for all litter types in the lab during early decay, but ratios increased to >1 during mid- and late decay. Field C:N and C:P ratios in mid-decay were >1. In the lab, C:N acquisition ratios were <1 for dogwood, maple, and oak, although the maple-oak mixture C:N and C:P ratios were >1 (**Figure 6B**). During late-decay, all litter types in the field had C:N and C:P ratios <1, but these patterns were reversed in the lab (**Figure 6C**). Overall, C:N and C:P ratios were >1 for all litter types in both studies (**Figure 6D**).

## DISCUSSION

### TEMPERATURE CONTROL OF MICROBIAL ACTIVITY

In contrast to expectations, mass loss during the initial 2 months of field decomposition (January–February) was <6%, even in the highly labile dogwood litter. We also found that enzyme activities and B:C ratios were lower than those found in later decay stages. Although temperature and moisture are strong controls on heterotrophic respiration (Schimel et al., 1994; Ise and Moorcroft, 2006; Suseela et al., 2012), field conditions during early decay included above-freezing soil temperatures (5.6°C), adequate moisture (32% WHC), and a substantial snowpack during most of February that minimized soil freezing. There was likely abundant labile C in fresh litter, and lower microbial demand for nutrients than C in all litters except oak (co-limited by C and P) suggests that either the high abundance of cellulose in fresh litter stimulated BG activity or N and P limitations were not acute. Additionally, greater mass loss for all litters under much lower moisture conditions during May through August 2010 suggests that field litter decomposition was not water-limited early in decay. Thus, we conclude that low temperatures suppressed decay rates during January–February. Temperature sensitivities are commonly reported to be inversely proportional to litter quality (Mikan et al., 2002; Fierer et al., 2006), with low temperatures inhibiting the degradation of easily available C polymers (Koch et al., 2007). For instance, Prescott (2010) proposed that microbial activity is uniformly low at temperatures below 10°C regardless of other factors, which is consistent with our findings, even with abundant labile C. In addition, it is known that the *Q*_10_ for respiration increases toward low temperatures and is about 4.5 at 10°C and 2.5 at 20°C (Kirschbaum, 1995, 2006). Thus, our finding that field decay rates were approximately 11 times lower than the lab during early decay is compatible with progressively higher *Q*_10_ toward lower temperatures, as suggested by developments in the understanding of how temperature affects microbial rates during decomposition (Kirschbaum, 2013).

### CARBON AND PHOSPHORUS EFFECTS ON MICROBIAL ACTIVITY

Under lab conditions, respiration peaked within the first week for all litter types, with the highest peak in dogwood. Dogwood has high concentrations of water-soluble compounds, cellulose, and nutrients (Moorhead and Sinsabaugh, 2000), and was the only litter type where microbial demand for C was greater than P during early decay (**Figure 6A**). It is possible that greater labile C availability in dogwood stimulated BG activity, as cellulose degradation increased more rapidly in dogwood than other litters during the first few days of decomposition (**Figure 4B**). However, litter types with high labile C concentrations often decompose rapidly when microorganisms are not P-limited because fast growing decomposers have proportionately higher P requirements (Gusewell and Freeman, 2005). Microbes were less efficient at hydrolyzing phosphate during early decay in our maple and oak litters (**Figure 5B**). Thus it is possible that higher dogwood P availability increased C mineralization rates and mass loss relative to more recalcitrant litters. These contrasts in demand for C and P between litter types suggest that litter quality and/or P availability influenced decomposer responses to leaf litter under temperature controlled conditions.

### ENVIRONMENTAL INFLUENCES ON MICROBIAL BIOMASS AND ENZYME DYNAMICS

Mass loss per unit enzyme activity was greater in the field than lab during mid-decay (**Figures 5C, D**). During this time, higher decay rates occurred in dogwood and maple under field than lab conditions (**Table 1**). Blair (1988) found that the total soluble content of dogwood and maple litter (>30% higher than oak) was the most important factor in first-year mass loss. This is likely due in part to the acceleration of mass loss by precipitation and leaching of soluble substrates. Decomposers also preferentially metabolize compounds in the soluble pool, as highly labile low-molecular weight substrates (i.e., sugars, phenolics, amino acids) are taken up with no enzymatic breakdown (Rinkes et al., 2011; Glanville et al., 2012). Thus, we speculate that the combination of leaching and microbial uptake of soluble compounds increased decay rates and mass loss per unit enzyme activity in the more labile litters under field conditions. It is unlikely leaching was as significant in microcosms because litter bags were placed in the middle of pre-wetted soil and only small amounts of water (1–2 ml) were added to the soil on a weekly basis to maintain moisture conditions. Thus, our findings suggest that environmental factors and litter soluble content influenced decay rates and mass loss per unit enzyme activity between studies during mid-decay.

It is also possible that greater faunal colonization and resulting fragmentation in the field increased the proportion of litter accessible to microbial attack and decreased the enzymatic effort needed to degrade dogwood and maple compared to more recalcitrant litters in the field (Cornelissen et al., 1999; Yang et al., 2012). For instance, lignified litters have high structural integrity that reduces soil faunal activity and fragmentation rates (Holdsworth et al., 2008). However, the exclusion of key soil macrofauna (e.g., non-native earthworms) due to the 1 mm^2^ mesh size of litter bags likely limited overall fragmentation in both studies compared to natural conditions. For example, litter decomposition rates are often more than eight-times greater in earthworm-accessible large mesh litter bags than in fine mesh bags that exclude them in temperate deciduous forests (Holdsworth et al., 2008; Fox et al., 2010). Therefore, we likely underestimated decay rates in both studies compared to natural conditions by excluding various soil faunal fragmenters.

Decomposer demand for N was greater than C in the lab for most litters during mid-decay (**Figure 6B**). Consistent with our mid-decay hypothesis, isolation from external nutrient subsidies and the limited amount of low nutrient soil used in the incubation likely reduced N availability and increased decomposer N limitation (Boberg et al., 2010). We also found higher BG activities (**Figures 4A, B**), but lower cellulose degradation per unit enzyme activity during mid-decay and overall in the lab than field for most litter types (**Figure 5**). It is possible that decomposers degraded soluble compounds and hemicellulose more rapidly under optimal lab conditions during early decay, especially in dogwood. If so, cellulose was a more important C source to decomposers in the lab after early decay. This likely stimulated BG activity, but decreased mass loss relative to the field. These differences in mass loss per unit enzyme activity and enzymatic effort directed toward C- and N- acquisition between decomposition studies suggest that both C and N availability constrain decomposer responses to litter quality.

Strong contrasts in mass loss per unit enzyme activity occurred between studies during late decay (**Figures 5E, F**). In addition, biomass to litter mass (B:C) ratios peaked in all litters during late decay in the field. However, B:C ratios declined between mid- and late decay in the lab (**Figure 2**). Coupled field and microcosm experiments demonstrate that field soils behave differently following disturbance (Teuben and Verhoef, 1992; Salamanca et al., 1998), primarily due to alterations in microbial community abundance, activity and composition. It is possible that soil collection decreased saprophytic fungal abundance (Coleman et al., 1988) and/or disrupted the N- and P-transporting mycelial network of mycorrhizae, likely increasing enzymatic effort to obtain nutrients from organic sources (Hart and Reader, 2004; Sheng et al., 2012). For instance, there is often an inverse relationship between total enzyme pool size and fungal biomass (Sinsabaugh et al., 2002). However, it is likely that hyphal transfer of nutrients by actinomycetes between soil and litter bags still occurred in both studies. Another potential explanation is that ${NO}_{3}^{-}$ accumulation (**Figure 3D**) suppressed ectomycorrhizal and saprotrophic fungal abundance (Carfrae et al., 2006; Wallenstein et al., 2006) and/or decreased overall microbial biomass in the lab (Ramirez et al., 2012). However, it is possible that high N throughputs occurred in the field as well, as our finding of consistently low field ${NO}_{3}^{-}$ concentrations are based on *in-situ* snap-shot measurements. Thus, our findings suggest relationships between microbial biomass and litter quality were not constant between the field and lab during late decay, likely due to differences in microbial community composition and/or N availability.

### THE INFLUENCE OF EDAPHIC FACTORS ON MICROBIAL FUNCTION

Oxidative enzyme activities only increased substantially in the high lignin oak litter and the maple-oak mixture in the field during late decay. Lignin is an aromatic polymer that is highly resistant to biological degradation, surrounds holocellulose in plant cell walls, and blocks microbial access to cell membrane proteins (Berg and McClaugherty, 2008). Thus, it is possible that a higher abundance of fungi specializing in lignin degradation in the field increased oxidative enzyme production in oak and the mixture to obtain protected labile N compounds (Talbot and Treseder, 2012). This is supported by our observation that microbial demand for N relative to C was greater for all litter types in the field after 40% mass loss (**Figure 6C**). Because the C return on investing in lignin degrading enzymes is low (Kirk and Farrell, 1987; Dashtban et al., 2010), our findings also suggest that decomposers likely degraded lignin to acquire shielded cellulose. For instance, the peak in BG activity was concomitant with the peak in oxidative enzyme activity in oak (**Figure 4**). Given that specialized fungal groups produce potent oxidative enzymes driving lignin degradation during later decay stages, an increase in their abundance and activity under field conditions likely influenced lignin degradation in our study (Bending and Read, 1997; Baldrian and Valaskova, 2008).

Oxidative enzyme activities never increased for any litter type in the lab. Consistent with our late-decay hypothesis, ${NO}_{3}^{-}$ accumulated in the lab (**Figure 3D**) and possibly decreased microbial demand for N relative to C across all litter types (**Figure 6C**). Although N can positively influence fungal colonization in fresh litter (Rousk and Baa, 2007), we speculate that excess N suppressed ectomycorrhizal and saprotrophic fungal activity during late-decay (Waldrop and Zak, 2006), which decreased lignin decomposition rates (Grandy et al., 2008). Additionally, the inability of lignin degrading fungi to colonize from adjacent litter in the lab probably limited lignin degradation, as spatial and temporal variations in fungal abundance and activity in field settings are common (Lindahl et al., 2007; Osono, 2007; Feinstein and Blackwood, 2013). It is also possible that cellulose was a more important resource than lignin to decomposers during late decay in the lab, which increased C:N acquisition ratios. For instance, mass loss was never greater than 50% for any litter type in microcosms, suggesting that unshielded cellulose may not have been completely depleted. Overall, however, our results suggest that decomposer isolation from external resources, and/or N inhibition of microbial activity decreased litter mass loss rates in the lab during late decay.

We found that mass loss of the maple-oak mixture was the average rate of the two individual litters, and hypothesize that this additive effect may be explained by N availability influences on microbial function. For instance, it is possible that the relative changes in microbial activity and decomposition rates of maple and oak due to changes in N availability were equal and the decomposition rate of the mixture was the average of the individual rates (Berglund and AA, 2012). Furthermore, unequal proportions of litter commonly result in non-additive effects (Mao and Zeng, 2012). Thus, the observed additive effect in our study was likely a result of N movement between the equal mixture of oak and maple litter used in our experiment (Schimel and Hattenschwiler, 2007).

### IMPLICATIONS FOR DECOMPOSITION MODELS

Our findings suggest that variable influences of climate and edaphic factors on microbial biomass, enzyme dynamics, and decomposition rates alter the trajectory of decay of varying leaf litter types. Although different microorganisms respond to temperature increases differently, our findings imply that microbial activity is predictably low across all decomposer groups below a temperature >5.6°C regardless of litter composition. Although not explicitly measured, we speculate that leaching and fragmentation increase access to soluble C compounds that do not require enzymatic hydrolysis prior to uptake, resulting in greater mass loss per unit enzyme activity in labile litter types. Additionally, our study demonstrates that N availability alters microbial responses to litter composition, with potentially strong effects on microbial abundance, activity, and enzymatic effort per unit mass loss in mid- and late decay. Different functional groups of decomposers may respond differently to N availability and target different substrates depending on their relative demand for C and N at different decay stages. Therefore, linking the N demands of different functional groups of decomposers to microbial behavior and decay rates of different litter substrates is likely to enhance the predictive capabilities of decomposition models.

## CONCLUSION

This study demonstrates that decomposition of the same leaf litter under field and lab conditions can result in strikingly different decay patterns due to the variable influences of climate and edaphic factors on microbial-substrate interactions. For instance, low temperatures decreased microbial activity early in decay, even in highly labile litters. These results in combination with previous findings of low microbial activity below 10°C and a higher *Q*_10_ at lower temperatures imply that even a small increase at lower temperatures may cause a substantial increase in CO_2_ flux. Furthermore, sharp contrasts in enzymatic effort directed toward C-, N-, and P-acquisition between litter types and studies indicate the importance of nutrient constraints on decomposer responses to litter quality. Finally, microbial biomass did not have a constant relationship to litter chemistry between the field and lab, which was likely caused by differences in nutrient availability and/or microbial community composition and function. Therefore, linking the C and N demands of different decomposer groups to decay rates of varying C substrates is likely to enhance mechanistic decomposition models.

## Conflict of Interest Statement

The authors declare that the research was conducted in the absence of any commercial or financial relationships that could be construed as a potential conflict of interest.
